# Genome-Wide Analysis of the Typical Thioredoxin Gene Family in Hexaploid Oil-Camellia: Identification, Phylogenetic Analysis, and Gene Expression Patterns

**DOI:** 10.3390/genes16070790

**Published:** 2025-06-30

**Authors:** Lan Wu, Peipei Song, Yifan Xia, Min Min, Tingting Xu, Junyong Cheng, Jihua Cheng, Huaguo Zhu

**Affiliations:** 1 College of Biology and Agricultural Resources, Huanggang Normal University, Huanggang 438000, China; w-lan@hgnu.edu.cn (L.W.); 18672652996@163.com (P.S.); 15826538139@163.com (Y.X.); mm676759669@163.com (M.M.); 15871359762@163.com (T.X.); 2 Hubei Key Laboratory of Economic Forest Germplasm Improvement and Resources Comprehensive Utilization, Huanggang Normal University, Huanggang 438000, China; 3 Hubei Academy of Forestry, Wuhan 430075, China; chengjunyong@163.com (J.C.); jihua.cheng@hotmail.com (J.C.)

**Keywords:** thioredoxins, *Camellia oleifera*, late flowering, gene expression

## Abstract

Hioredoxins are small proteins crucial for maintaining cellular redox balance and are involved in various biological processes, including growth, photosynthesis, development, and stress responses. This study aims to conduct a genome-wide analysis of the typical Thioredoxin (TRX) gene family in hexaploid *Camellia oleifera* and explore the role of the CoTRX25 gene in flowering. Through bioinformatics approaches, we identified 27 typical TRX gene family members in the *C*. *oleifera* genome and analyzed their phylogenetic relationships, gene structures, conserved motifs, and chromosomal distributions. Transcriptomic analysis across different tissues was performed to determine the expression patterns of these genes. Additionally, the *CoTRX25* gene was cloned and heterologously overexpressed in *Arabidopsis thaliana* to investigate its functional role in flowering. The 27 TRX genes were mainly located on 11 chromosomes, with multiple gene duplication events identified, indicating that gene duplication has played a significant role in the expansion of the TRX family. Transcriptomic analysis revealed that most typical TRX genes are highly expressed in embryos, suggesting their potential importance in seed development. Overexpression of *CoTRX25* in *A. thaliana* led to delayed flowering, implying that this gene may be involved in flowering regulation. This study provides a theoretical basis for understanding the functions of typical TRX genes in *C. oleifera* growth and development, particularly highlighting the role of *CoTRX25* in flowering regulation.

## 1. Introduction

*Camellia oleifera* is a plant species native to southern China with significant economic value. It is well-known for its high-quality oil extracted from seeds, which is rich in monounsaturated fatty acids, especially oleic acid [1,2]. This oil is highly regarded for its nutritional and health benefits, comparable to those of olive oil [3]. The seeds of *C. oleifera* are not only a crucial source of edible oil but also hold potential for the development of functional foods and pharmaceuticals [4].

Thioredoxins (TRXs) are essential small proteins that play a vital role in maintaining cellular redox homeostasis and are involved in a variety of biological processes, such as cellular growth, photosynthesis, development, and stress responses [5,6]. The TRX family is divided into two main categories: typical and atypical TRXs [7]. Typical TRXs, characterized by the conserved WCGPC in their active sites, are particularly diverse and evolutionarily significant in plants, participating in numerous redox-dependent reactions [7,8]. This category encompasses several subfamilies, including H, F, M, X, Y, Z, O, and TDX, which are typically reduced by TRX reductases [9]. In contrast, atypical TRXs form the TRX-like subfamily and contain distinct active sites such as WCRKC and WCRVC [7,10]. In the realm of photosynthesis, typical TRXs have emerged as key regulators. They facilitate the light activation of ADP-glucose pyrophosphorylase, an enzyme crucial for starch synthesis, thereby playing a significant role in diurnal starch turnover in leaves [11]. In spinach, the TRX-F subtype is known for its role in catalyzing fructose-1,6-bisphosphatase (FBPase), and its deficiency in *A. thaliana* mutants result in impaired growth and photosynthetic activity under fluctuating light conditions [11]. Additionally, TRX-M proteins, another subgroup of typical TRXs, are involved in regulating cyclic electron transport in chloroplast photosystem I, which is essential for managing light energy and protecting the photosynthetic apparatus from overexcitation [12]. Beyond their role in photosynthesis, typical TRX family genes are also integral to the plant’s antioxidant response system. They help mitigate oxidative stress caused by environmental challenges such as drought, high temperature, and salinity [5]. TaTrxh, a typical TRX, has been associated with heading-time regulation in wheat. CRISPR-Cas9-mediated gene knockout studies have confirmed that the TaTrxh9 mutation leads to early heading [13]. The typical TRX family genes are essential components of the plant’s photosynthetic machinery and stress response system.

The genome-wide analyses of the TRX family have been widely carried out because the genomes of many species have been sequenced in recent years. In total, 41, 61, 48, and 15 TRX family members have been identified in *Arabidopsis* [7], rice [14], *Liriodendron* [15], and upland cotton [16], respectively. The TRX family in *C. oleifera* has attracted much attention due to its potential roles in enhancing stress tolerance and optimizing metabolic pathways [17]. However, due to the complexity of the camellia oil genome, the current research on the TRX family genes is incomplete. In this study, we conducted genome-wide identification and multifaceted analyses of typical TRX family members in hexaploid *C. oleifera* genome using domain and protein sequences, gene structures, phylogenetic relationships, synteny, and gene expression profiles from different tissues. Additionally, we cloned the *CoTRX25* from the genome, explored its functions heterologous overexpression in *A. thaliana*. This study provides a basis for effectively understanding the roles of typical CoTRX members in *C. oleifera*.

## 2. Materials and Methods

### 2.1. Genome-Wide Identification of TRXs Genes in the Camellia oleifera Genome

To identify TRX genes in *C*. *oleifera*, we utilized the chromosome-level genome of *Camellia oleifera* var. Changlin40 [18] and performed a search using *Arabidopsis* TRX sequences obtained from Kim et al. [19]. The search was conducted via NCBI BLASTP with parameters set at an E-value of less than 1 × 10^−20^ and a maximum of five target sequences. The coding DNA sequence (CDS), protein sequence, and general feature format version 3 (GFF3) files for the *C*. *oleifera* var. Changlin40 genome were retrieved from the figshare database (https://figshare.com/s/1a51c1909eab9cc0b603, accessed on 29 May 2024). Additionally, the hidden Markov model (HMM) for TRX (PF00085) was obtained from the Pfam database (http://pfam.xfam.org/, accessed on 29 June 2024). Using the HMMER 3.1 search tool, we screened for TRX proteins by setting the cutoff value for the HMM search program at 0.001. The putative CoTRX candidates were then submitted to the NCBI CDD server (https://www.ncbi.nlm.nih.gov/cdd/, accessed on 30 June 2023), SMART database (http://smart.embl.de/, accessed on 1 January 2023), and HMMscan (https://www.ebi.ac.uk/Tools/hmmer/search/hmmscan/, accessed on 1 August 2023) to confirm the presence of the TRX domain. Finally, proteins containing the “WCGPC” sequence motif within the TRX domain were selected. These proteins were named based on their chromosomal locations. In total, 27 CoTRX genes were identified in this study.

### 2.2. Analysis of Conserved Motifs and Structural Domains of Typical TRX Protein in Camellia oleifera

The conserved motifs of typical TRX proteins were predicted and analyzed using the MEME website (https://meme-suite.org/meme/doc/meme.html, accessed on 1 March 2024), with the motif number set to 10 while other parameters were kept at their default values. The analysis result file was downloaded with the file name mast.xml. Based on the whole genome annotation file, the analysis was performed to obtain the exact position and number information of exons and introns of typical TRX in oil tea. TBtools software (V.2.114) was used to draw a comprehensive map of the conserved motifs, structural domains, and evolutionary trees of CoTRXs proteins [20].

### 2.3. Protein Sequence Analysis and cis-Regulatory Elements Analysis of Typical CoTRX Promoters

Protein subcellular localization of CoTRX proteins were predicted by webserver BUSCA (http://busca.biocomp.unibo.it/, accessed on 1 November 2023). The possible tertiary structures of CoTRXs were predicted by Phyre2 web portal [21]. The 2 Kb sequences upstream of the start codon ATG site of *CoTRXs* were considered putative promoters. cis-Regulatory elements in these putative promoters were predicted by PlantCARE website [22]. The physical and chemical parameters of CoTRXs, including molecular weight (MW), isoelectric point (PI), and the number of amino acids and the exon-intron structures and domains of *CoTRXs* were both visualized by using TBtools [20].

### 2.4. Phylogenetic and Molecular Evolution Analyses

CoTRX polypeptide sequences of self-identified (*Camellia sinensis* var. *sinensis*) and published (*Arabidopsis thaniana*, *Oryza sativa*) from genome database (https://phytozome-next.jgi.doe.gov/, accessed on 1 August 2023) (Table S1). A phylogenetic tree of TRXs between the tea-oil tree and *Arabidopsis* was reconstructed using maximum likelihood by MEGA 11.0 with optimal amino acid substitution model of JTT + G4. The phylogenetic relationship of TRXs among 4 land plant species was reconstructed using the neighbor-joining algorithm by MEGA 11.0 with optimal amino acid substitution Dayhoff + G1 model. All trees were estimated using 1000 bootstraps with the parameter of -bb 1000. The online tool of iTOL was used for the display of TRX trees. Duplicate gene pairs of *CoTRXs* were characterized by MCscanX. TBtools was used for molecular evolutionary analyses of the *CoTRX* genes, which were performed by calculating the nonsynonymous (*Ka*) to synonymous (*Ks*) substitution rate ratios using the KaKs_Calculator online tool, and for visualizing the results of collinearity analyses within *CoTRX* genes [20].

### 2.5. Gene Expression and Protein–Protein Interaction Network Analyses

Per million mapped reads (FPKM) values of *CoTRXs* in various tissues, including the stems, leaves, flower buds, embryos, and seeds, were obtained in our laboratory (NCBI accession number: PRJNA993817). Using this transcriptome data, we examined the expression patterns of *C*. *oleifera* TRXs from seed kernel samples at six developmental stages (220, 240, 260, 280, 300, and 320 DAP, day after pollination). Three independent biological replicates were collected for each tissue/developmental stage. A heatmap of *CoTRXs* was generated by TBtools and the gene expression was estimated by the log-transformed FPKM (log2[FPKM]) values. The protein–protein interaction networks among CoTRXs were predicted and displayed by STRING database v12.0 (https://string-db.org/, accessed on 1 January 2023).

### 2.6. Gene Cloning and Transgenic Plant Construction

The full-length coding sequence of the *CoTRX25* gene was amplified via PCR using specific primers (Table S2). The overexpression vector of CoTRX25 was constructed using homologous recombination with KpnI (TRANSgene, Beijing, China) single-enzyme digestion, utilizing the pCAMBIA 1300 vector to construct the 35S:CoTRX25 overexpression construct. Transgenic *Arabidopsis* plants overexpressing *CoTRX25* were generated via *Agrobacterium*-mediated transformation using strain GV3101, with empty vector transformed plants serving as controls. Homozygous 35S:CoTRX25 lines were obtained through the floral dip method [23] and selected according to Ye et al. [24]. Transgenic plants were selected on MS medium supplemented with 100 mg/L kanamycin. After 14 days of selection, resistant seedlings were transferred to commercial nutrient soil and cultivated in a growth chamber under controlled conditions as follows: a 16 h/8 h (light/dark) photoperiod at 25 °C, with relative humidity maintained at 50–70%.

## 3. Results

### 3.1. Genome-Wide Identification and Physicochemical Properties of Typical TRX in Hexaploid C. oleifera Genome

**Figure 1:**
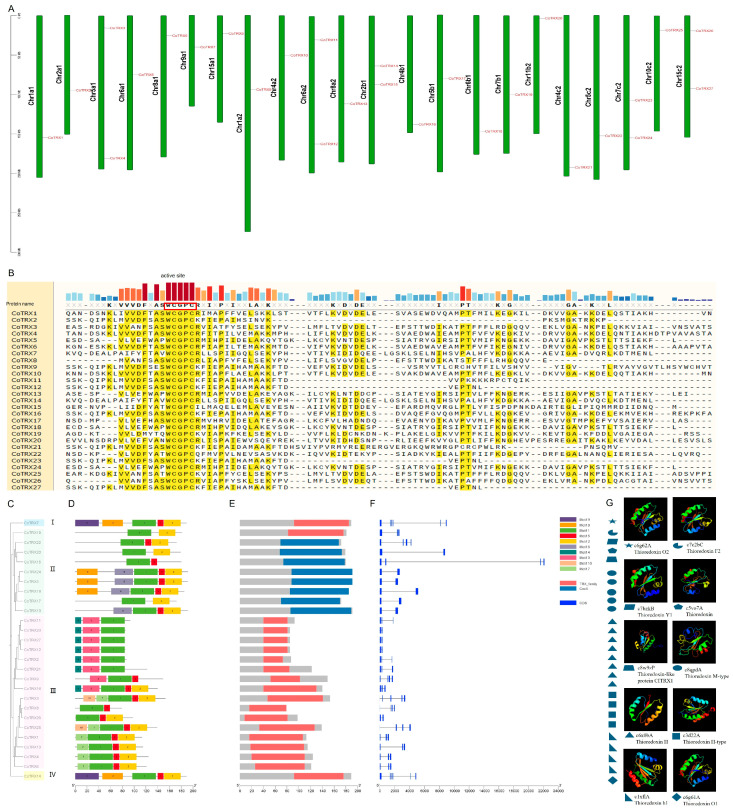
Phylogenetic relationships, gene structure, and conserved motifs of typical CoTRX gene members. (**A**) Chromosomal locations and distributions of the identified CoTRX genes. (**B**) Alignment of consensus protein sequences of typical CoTRXs which contained the active-site Cys residues and the catalytic motif are indicated by ‘WCGPC’ (framed with rectangle). (**C**) Maximum likelihood-based phylogenetic tree of CoTRXs. (**D**) The characterization of the putative motifs in typical CoTRX proteins. (**E**) The characterization of the CDDs in typical CoTRX proteins. (**F**) The exon–intron structure of typical CoTRX genes. Differently colored boxes represent various types of conserved motifs. (**G**) Predicted tertiary structures of CoTRX sequences.

In the retrieved CoTRX protein sequences, proteins containing the “WCGPC” sequence in the TRX domain were screened, and 27 full-length genes encoding putative typical TRX gene family members were finally identified. They were named *CoTRX1* to *CoTRX27* according to the chromosome order on which they were located and the highest expression level among haplotypes (Figure 1A). The active-site Cys residues and a redox-active disulfide motif (WCGPC) were identified by multiple sequence alignment analysis of consensus sequences (Figure 1B).

The phylogenetic relationship of typical CoTRXs was constructed using the amino acid sequences (Figure 1C). The phylogenetic tree showed that typical CoTRXs were clustered into four main classes which were termed Class I to IV in this study with 100% bootstrap support. Typical CoTRXs can be further grouped into seven clades (F type, H type, M type, O type, X type, Y type, and Z type), and all these except TDX contain more than one member. A total of 9 out of 27 of the typical CoTRXs were classed into Class II, and 16 out of 35 members belonged to Class III. Pairwise comparison of full-length TRX protein sequences showed that pairwise identity scores of all Class II TRXs was 44.81%, pairwise identity scores of all Class III TRXs was 34.11%, suggesting a relatively higher sequence similarity within Class II.

Subcellular localization prediction of 27 typical CoTRX showed that these proteins may be localized in 8 different cellular compartments, including chloroplast (12), chloroplast outer membrane (5), chloroplast thylakoid membrane (1), cytoplasm (2), endomembrane system (1), extracellular space (2), mitochondrion (1), and nucleus (3). Predicted tertiary structures of typical CoTRX sequences were modeled 10 with templates based on the protein fold recognition server PHYRE2 (Figure 1G, Table 1). Predicted tertiary structures of eight of CoTRXs were similar to c6x0bA and five TRXs were similar to c8qpdA. While four and four TRXs were similar to c1xflA and c3d22A, only one TRXs were similar to c5vo7A, c6g61A, c6g62A, c7bzkB, c7c2bC. and c8w9zP, respectively (Figure 1G). The results revealed that Class II TRXs belonged to Thioredoxin M-type, while the Class III PGs were Thioredoxin H-type. Generally, closely related genes usually had common gene structures (Figure 1D–G).

The lengths of predicted CoTRXs proteins ranged from 79 to 191 amino acid (Table 1). The proteins of the family members are composed of at least 79 amino acids (CoTRX8), and at most 191 amino acids (CoTRX5 and CoTRX13). The molecular weights (MWs) of this family of proteins ranged from 9.09 kDa (CoTRX8) to 21.15 kDa (CoTRX7). The isoelectric points (pIs) ranged between 4.6 (CoTRX8) and 9.96 (Co-TRX24), of which 13 family members had pIs greater than 7, and 14 family members had pIs less than 7. According to the average coefficient of hydrophilicity, of which 18 family members had negative GRAVY values, indicating that these proteins were hydrophobic (GRAVY), while 9 family members had positive GRAVY values, indicating that these proteins were hydrophilic.

### 3.2. Evolutionary Analysis of Typical TRX Gene Family in Camellia oleifera

**Figure 2:**
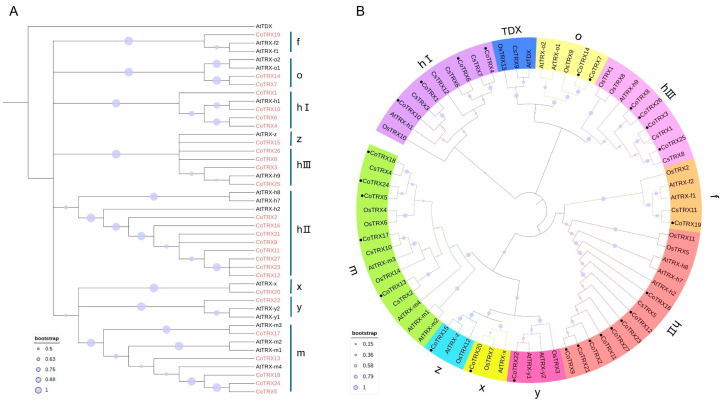
Phylogenetic relationship of TRXs. (**A**) Phylogenetic tree of the *C. oleifera* and *Arabidopsis* TRXs. (**B**) Phylogenetic tree of TRXs from the *C. oleifera* and 4 other species.

A rooted phylogenetic tree including 27 CoTRXs and 18 *Arabidopsis* TRXs was reconstructed with the maximum likelihood (ML) method using AtTDX as the outgroup (Figure 2A). None of the CoTRXs were clustered into TDX. Phylogenetic relationship among 71 typical TRXs from 4 species was also analyzed in this study. Altogether, we identified 14, 18, 12, and 27 TRX genes from rice, *Arabidopsis*, tea tree, and *C. oleifera* genomes, respectively (Table S1). The number of typical TRX family members in *C.oleifera* was approximately 2-fold of that in rice and tea tree. Intriguingly, according to the NJ tree reconstructed from amino acid sequences, the typical TRXs were classified into 8 groups, corresponding to h, m, f, o, x, y, z, and TDX. Among them, h type had the largest members and divided into hI (10), hII (14), and hIII (9) members (Figure 2B). All species studied, which included monocots and eudicots, had TDX genes except for the *C*. *oleifera*, suggesting that there were several possible gene losses during the evolution of them.

### 3.3. Collinearity and Selective Pressure Analysis

**Figure 3:**
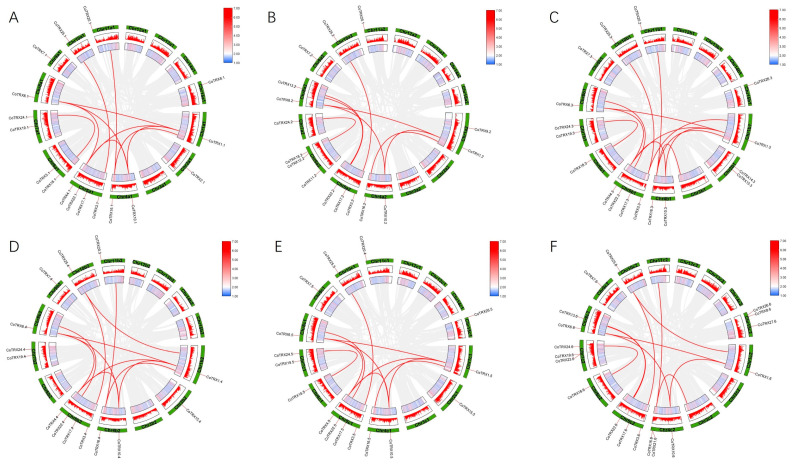
Genomic distribution and collinearity analysis of typical *CoTRXs* family members. The red lines represent collinearity relationships of the paralog *CoTRXs*. The gray lines represent collinearity relationships in *C. oleifera* genome. (**A**–**F**) represent the 6 haplotypes from chra1 to chrc2 respectively.

The distribution of typical *CoTRXs* on *C. oleifera* chromosomes was examined across six different haplotypes. All typical *CoTRXs* were dispersed on 11 chromosomes (Figure 3), while the number of typical *CoTRXs* on each haplotype ranged from 14 to 18 (Table S3). Among them, c2 had the highest count, with 18 genes; a1 and b1 each possessed 17 genes; a2 and c1 each contained 16 genes; and b2 included only 14 genes. Altogether, the most homologous genes are distributed on four (*CoTRX10/16/21*), five (*CoTRX3/4/17/22*) and seven (*CoTRX19/23/24*) chromosomes. Each haplotype contains TRX genes that have corresponding orthologous genes in the other haplotypes, indicating a high degree of conservation during evolution. Gene duplications of 27 *CoTRXs* were investigated by MCScanX. The *CoTRXs* presented the characteristics of cluster distribution on chromosomes 1a1, 2a1, 6a1, 8a1, 9a1, 15a1, 1a2, 4a2, 8a2, 4b1, 5b1, 6b1, 7b1, 11b2, 4c2, 5c2, and 10c2. Eight pairs of *CoTRXs* (*CoTRX1.1*/*4.1*, *CoTRX1.1*/*6.1*, *CoTRX1.1*/*10.1*, *CoTRX3.1*/*25.1*, *CoTRX4.1*/*10.1*, *CoTRX4.1*/*6.1*, *CoTRX6.1*/*10.1*, *CoTRX18.1*/*24.1*) were arranged in segmental duplications on chromosomes a1, while the other eight pairs were dispersed on this chromosome. Only two pairs of *CoTRXs* (*CoTRX3.2*/*25.2*, *CoTRX18.2*/*24.2*) were arranged in segmental duplications on chromosomes a2. Eight pairs of *CoTRXs* (*CoTRX1.3*/*4.3*, *CoTRX1.3*/*6.3*, *CoTRX3.3*/*25.3*, *CoTRX4.3*/*6.3*, *CoTRX4.3*/*10.3*, *CoTRX18.3*/*24.3*) were arranged in segmental duplications on chromosomes b1, while the other seven pairs were dispersed on this chromosome. Five pairs of *CoTRXs* (*CoTRX1.4*/*4.4*, *CoTRX1.4*/*6.4*, *CoTRX1.4*/*10.4*, *CoTRX3.4*/*25.4*, *CoTRX4.4*/*6.4*) were arranged in segmental duplications on chromosomes b2, while the other six pairs were dispersed on this chromosome. Seven pairs of *CoTRXs* (*CoTRX1.5*/*4.5*, *CoTRX1.5*/*6.5*, *CoTRX1.5*/*10.5*, *CoTRX3.5*/*25.5*, *CoTRX4.5*/*10.5*, *CoTRX6.5*/*10.5*, *CoTRX18.5*/*24.5*) were arranged in segmental duplications on chromosomes c1, while the other five pairs were dispersed on this chromosome. Six pairs of *CoTRXs* (*CoTRX1.6*/*6.6*, *CoTRX1.6*/*10.6*, *CoTRX3.6*/*25.6*, *CoTRX6.6*/*10.6*, *CoTRX13.6*/*22.6*, *CoTRX18.6*/*24.6*) were arranged in segmental duplications on chromosomes c1, while the other eight pairs were dispersed on this chromosome. Overall, *CoTRX3*/*25* and *CoTRX18*/*24*, associated with segmental duplications, were distinguished based on conserved collinearity linkage in all haplotypes (Figure 3).

The nonsynonymous substitution rate (*K_a_*), synonymous substitution rate (*K_s_*), and *ω* (*K_a_*/*K_s_*) of *CoTRX* paralog pairs were calculated (Table 2). The results showed that the *K_s_* values of segmental duplication pairs of *CoTRXs* (ranging from 0.2685 to 1.4789) were smaller than dispersed pairs (ranging from 0.0380 to 5.8251). The *ω* values of all *CoTRX* paralog pairs were less than 1 (ranging from 0.1416 to 0.4237), suggesting strong negative selection on duplicated *CoTRXs*.

### 3.4. cis-Regulatory Element Analysis of Typical CoTRX Promoters

**Figure 4:**
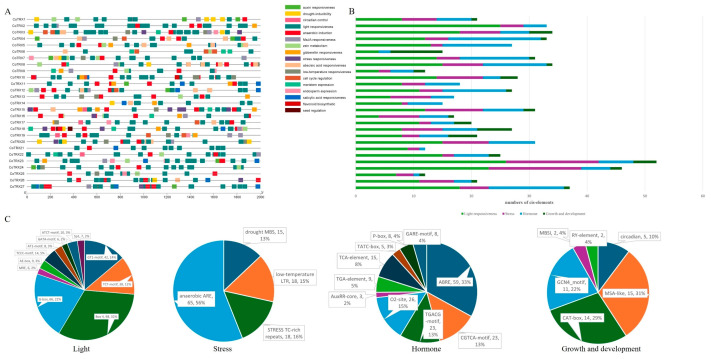
cis-Regulatory elements analysis of promoters of typical *CoTRXs*. (**A**) Numbers of each cis-regulatory elements in the promoter region of each *CoTRX*. (**B**) The stacked column chart of cis-regulatory elements related to light responsive, stresses, hormone responsive, and plant growth and development. (**C**) Pie charts of four groups of cis-regulatory elements.

A total of 648 cis-regulatory elements associated with light responsiveness (304), stresses (116), hormone responsiveness (179), and plant growth and development (49) were identified from 2 kb region upstream of the start codon ATG site of typical *CoTRXs* (Figure 4). All *CoTRXs* contained at least one light response element, including GT1-motif, TCT-motif, TCCC-motif, GATA-motif, and Sp1 which are involved in light responsive element, AT1-motif, ATCT-motif, Box 4, and AE-box which are parts of a module involved in light responsiveness, G-box which is a regulatory element involved in light responsiveness, MRE which is involved in MYB binding site involved in light responsiveness. All *CoTRXs* contained hormone-responsive elements which were associated with responses to gibberellin (GA; TATC-box, GARE-motif, and P-box), abscisic acid (ABA; ABRE element), methyl jasmonate (MeJA; TGACG-motif/CGTCA-motif), salicylic acid (SA; TCA-element), and zein (O2-site). Apart from *CoTRX6*, all *CoTRXs* contained cis-elements associated with stress which were related to drought (MYB binding site element, MBS), low-temperature responsiveness (low-temperature-responsive element, LTR), defense and stress responsiveness (TC-rich repeats) and anaerobic induction (anaerobic responsive element, ARE). A total of 20 of the 27 TRX members were found to possess cis-acting elements linked to growth and development (Figure 4A). The MYB binding site, which is involved in flavonoid biosynthetic genes regulation (MSBI) and seed-specific regulation element (RY-element), only existed in two promoters of *CoTRXs*. GCN4_motif, which is related to endosperm expression, existed in nine promoters. The MSA-like element, which is related to cell cycle regulation, existed in eight promoters. CAT-box element (GCCACT), which is related to meristem expression, existed in seven promoters. The Circadian element, which is related to circadian control, existed in five promoters.

### 3.5. Gene Expression and PPI Network Analysis of Typical CoTRXs

**Figure 5:**
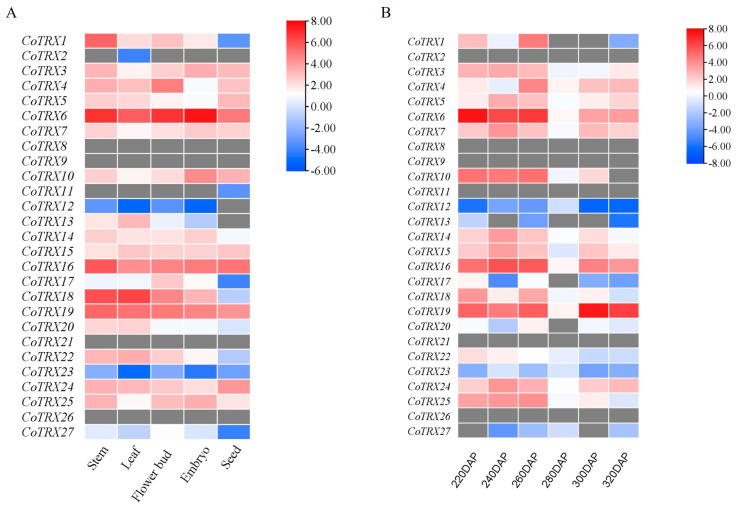
Gene expression trend and qRT-PCR validation of *CoTRXs*. (**A**) Expression levels of 27 typical *CoTRX* genes based on FPKM values in 5 tissues. (**B**) Expression levels of typical *CoTRX* genes based on FPKM values during embryo development.

The spatiotemporal-specific expression clustering heatmap was plotted based on log2 FPKM values in stems, leaves, flower buds, embryos, and seeds (Figure 5A and Table S4). Among the typical *CoTRXs*, over 74% (20 out of 27) exhibited expression in at least one tissue, with *CoTRX2/8/9/11/12/21/26* being the exceptions. Nine *CoTRXs*, including *CoTRX3/6/7/10/15/16/19/24/25*, were expressed in all above tissues (Figure 5A). Most of the homologous genes of typical *CoTRXs* had similar spatiotemporal expression patterns; h type (highly expressed in stems), m type, f type, x type, and y type were highly expressed in the stems and leaves, while o type was highly expressed in embryos and stems. However, some homologous genes showed different expression patterns. For example, *CoTRX17* (m type) and *CoTRX27* (hII type) were highly expressed in the flower buds, while *CoTRX25* (hIII type) was highly expressed in the stem, flower buds and embryo, suggesting that these homologous genes were subfunctionalized. Compared with stems (27), only a small number of *CoTRXs* expressed in leaves (17), flower buds (17), embryos (14), and seeds (11), suggesting their potential regulatory roles in multiple developmental processes, and *CoTRXs* of embryos had the highest expression levels than other tissues. The expression profiles of *CoTRXs* in *C. oleifera* embryos were further explored. A total of six different development stages were identified (Figure 5B; Table S4). During embryo development, almost all genes showed similar expression patterns, with two peaks expression and one nadir expression. A total of 11 out of 27 typical *CoTRXs* had the expression profile that peaks of expression at 260DAP (260 day after pollination) and 300DAP, nadir of expression at 280DAP. *CoTRX5*/*13*/*24* (m type), *CoTRX7*/*14* (o type), and *CoTRX15* (z type) were highly expressed at stages of 240DAP and 300DAP and nadir of expression at 280DAP in seed kernel samples. *CoTRX12*/*13*/*23* showed different expression patterns in the 260DAP trough, 280DAP peaks may suggest that these *CoTRXs* were highly correlated with the seed kernel development.

**Figure 6:**
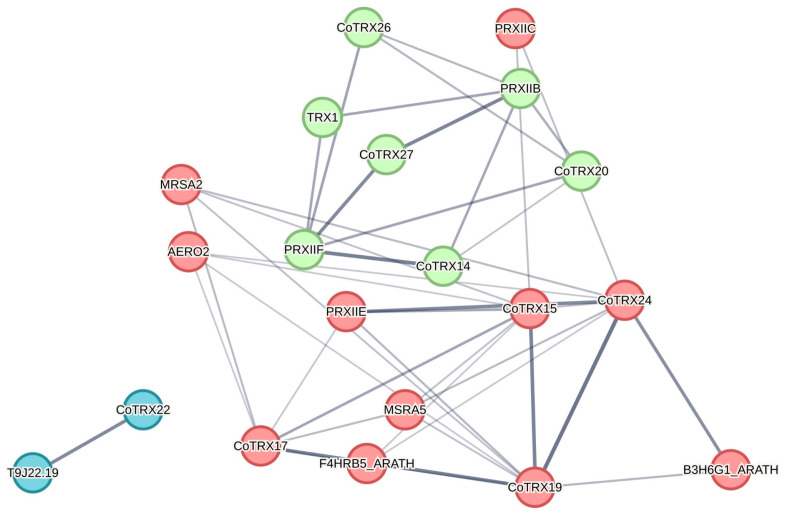
Protein–protein interaction networks predicted by STRING. Line thickness indicates the strength of data support. The gene names of *Arabidopsis* were marked with blue color.

The protein–protein interaction networks among CoTRXs were predicted by the STRING database based on the interacting protein pairs in *Arabidopsis* (Figure 6). The network was clustered into three clusters by k-means. Nine interactive relationships between CoTRXs were predicted. CoTRX15 (CITRX) and CoTRX20 (ATHX) were possibly most widely interacted with other CoTRXs, and then with CoTRX14 (TO1), CoTRX17 (GAT1-2), CoTRX19 (TRXF2), CoTRX24 (TRX-M4), and CoTRX26 (TRX9). CoTRX26 (TRX9) may only interact with CoTRX20 (ATHX). CoTRX17 (GAT1-2) may only interact with CoTRX15 (CITRX). And CoTRX14 (TO1) may interact with CoTRX15 (CITRX) and CoTRX20 (ATHX). They were reported to be involved in the redox regulation of enzymes of both Calvin–Benson cycle and stress defense responses.

### 3.6. Overexpression of CoTRX25 Leaded to Late Flowering of Transgenic Arabidopsis

**Figure 7:**
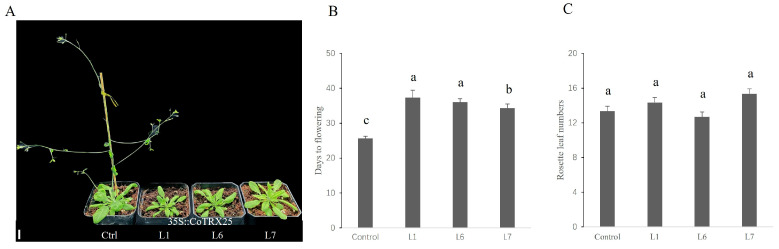
Ectopic overexpression of *CoTRX25* in *Arabidopsis* caused late flowering. (**A**) Phenotype of *Arabidopsis* control and *CoTRX25* transgenic plants grown under long-day (16 h light/8 h dark) conditions in a growth chamber. Scale bar, 1 cm. (**B**) The days to flowering as determined by the day floral buds became visible. Letters denote significantly different groups (ANOVA, *p* < 0.05). (**C**) Rosette leaf numbers of vector control and *CoTRX25* transgenic *Arabidopsis* plants. Letters denote significantly different groups (ANOVA, *p* < 0.05).

To elucidate the functional role of *CoTRX25* in plant growth, both control and overexpression transgenic T2 homozygous plants were obtained. The overexpressed plants showed late flowering compared to the control (Figure 7A). Flowering times were recorded for three representative plants from each of the control (25.7 ± 0.6 d), Line 1 (37.3 ± 2.1 d), Line 6 (36.0 ± 1.0 d), and Line 7 (34.3 ± 1.3 d) (Figure 7B). No significant difference was observed in the number of rosette leaves between the control and transgenic lines (Figure 7C). These results highlight their involvement in flowering time regulation and expand the functional diversity of TRX genes.

## 4. Discussion

The completion of whole genome sequencing and genome structure annotation accelerates the process of deciphering the genetic code of plant species. Using the chromosome-level reference genome of hexaploid *C. oleifera* [18], the origin, evolution, and gene function of various gene families involved in key biological processes (such as lipid biosynthesis, stress response, and growth regulation) of *C. oleifera* can be explored with high efficiency through bioinformatics methods. At present, some important gene families which are closely related to disease resistance and seed oil biosynthesis, such as the WRKY gene family, MADS-Box Genes, and Dof gene family, have been reported in *C. oleifera* [25,26,27]. *TRXs* are involved in redox regulation of a wide variety of processes, such as plant growth and development, and alleviating oxidative stress [5,6]. However, typical TRX family genes in *C. oleifera* have not been identified yet.

In this study, based on combined analyses of sequence similarity and domain information, a total of 27 typical *CoTRXs* which were randomly distributed on 22 chromosomes were identified and mainly classified into 4 classes which were further grouped into 7 clades by reconstruction of phylogenetic relationships (Figure 1 and Figure 2). The number of genes were differed from that of *Arabidopsis* (18), wheat (*Triticum aestivum*) (48), cotton (*Gossypium hirsutum*) (40), and tea (*C. sinensis*) (12) [7,16,17]. These subgroups vary in the number of genes and active site sequences, suggesting that these orthologous pairs might have existed before the differentiation of monocotyledon ancestors. The expansion of certain subgroups may represent an evolutionary adaptation to environmental changes, and gene duplication appears to be species-specific. The absence of the TDX subfamily in *C. oleifera* is notable, as this subfamily is present in other plants such as *A. thaliana*, *O. sativa,* and *G. hirsutum* [7,14,16]. This suggests that *C. oleifera* may have experienced homologous copy loss events during its polyploid evolution. This pattern of gene loss may reflect selective pressures acting on the genome, potentially related to functional redundancy or specific adaptations to environmental conditions.

Furthermore, our findings indicate that gene duplication, particularly segmental duplications (SDs), has played a significant role in shaping the typical TRX gene family in *C*. *oleifera* (Table 2). This is consistent with observations in other species, such as *T. aestivum* (wheat), where segmental duplications have contributed to the expansion and functional diversification of gene families [17]. SDs could extend beyond the coding portion of the barley genome and play a fundamental role in shaping copy number variants (CNVs) [28]. Segmental duplications can provide additional genetic material for evolutionary adaptation and functional innovation, which may be particularly important in polyploid species where genetic redundancy can buffer against deleterious mutations [28,29]. The combination of gene loss and segmental duplications in *C. oleifera* highlights the dynamic nature of polyploid genomes and underscores the importance of understanding the evolutionary forces shaping gene family diversity.

The high expression of CoTRX genes (*CoTRX6*, *CoTRX10*, *CoTRX16*, *CoTRX18*, and *CoTRX19*) in *C. oleifera* embryos (Figure 5) indicates their crucial roles in embryonic development. This aligns with findings in *A*. *thaliana* and *O*. *sativa*, where TRX genes are highly expressed in embryos and play key roles in redox regulation and stress response during embryogenesis [7,8]. However, specific TRX genes and their expression patterns vary across species. For instance, *AtTRXh1* and *AtTRXh2* in *Arabidopsis* primarily regulate redox processes [7]. Similar diversity is observed in *Citrus sinensis*, where some CsTRX genes (e.g., *CsTRXf1*, *CsTRXh1*) show reduced expression upon Huanglongbing (HLB) infection, highlighting their potential roles in plant immune responses [30]. In *Liriodendron chinense*, *LhTRX*-*h3* was upregulated under drought stress, enhancing drought tolerance by reducing ROS accumulation [15]. These findings underscore the conserved yet species-specific functions of TRX genes in embryonic development and stress responses. In *C*. *oleifera*, *CoTRX25* (m-type TRX) acts as a flowering inhibitor, contrasting with *GhTRXL3*-*2* (atypical TRX) in *G. hirsutum* which promotes flowering [16], and TaTrxh9 (h-type TRX) in wheat that accelerates heading when suppressed [13]. These functional divergences likely reflect both species-specific adaptations to distinct ecological constraints and subfamily specific functional specialization. While GhTRXL3-2 promotes flowering through direct interaction with GhFT in the FAC pathway [16], *TaTrxh9* regulates heading time through redox-mediated signaling [13]. The flowering in *C. oleifera* is governed by the integration of hormone signaling (IAA, GA, JA) and circadian clock components (*GI*/*CO*), which converge on *FT*/*SOC1* to activate *LFY*/*AP1* and drive floral transition [31]. Notably, we identified *CoTRX25* as a potential new flowering regulator in the TRX gene family through an unknown mechanism in *C. oleifera*. This finding expands our understanding of TRX-mediated flowering regulation and highlights species-specific adaptations in perennial plants. Future studies should explore the functional mechanisms of *CoTRX25* and its potential interactions within the flowering regulatory network of *C. oleifera*.

## 5. Conclusions

This study comprehensively characterizes the typical TRX gene family in hexaploid *C. oleifera*, identifying key genes such as *CoTRX25* that delay flowering when overexpressed in *Arabidopsis* and *CoTRX6*, *CoTRX10*, *CoTRX16, CoTRX18*, and *CoTRX19* which are highly expressed in embryos and likely crucial for early embryonic development. These findings provide a framework for further investigation of oil biosynthesis and optimizing flowering time in *C*. *oleifera*. Future work should focus on validating these roles through genetic engineering techniques and exploring their broader applications in agricultural biotechnology.

## Figures and Tables

**Table 1:** Summary of typical TRX proteins identified in hexaploid *Camellia oleifera*.

Gene Name	Locus ID	Chromosome Number	Predicted Size (aa)	Molecular Weight (kDa)	Isoelectric Points (pI)	Exon	Subcellular Localization Prediction	Tertiary Structure Prediction Template Protein Data Bank Molecule	
*CoTRX1*	A1.1G231500	chr1a1	113	12.51	5.51	3	Nucleus	c1xflA	Thioredoxin h1
*CoTRX2*	A1.2G146400	chr2a1	87	9.50	9.83	3	Chloroplast	c6x0bA	Thioredoxin H
*CoTRX3*	A1.5G005400	chr5a1	153	17.25	6.07	5	Chloroplast	c3d22A	Thioredoxin H-type
*CoTRX4*	A1.5G295300	chr5a1	124	13.67	5.29	3	Cytoplasm	c1xflA	Thioredoxin h1
*CoTRX5*	A1.6G124800	chr6a1	191	20.64	9.36	2	Chloroplast outer membrane	c8qpdA	Thioredoxin M-type
*CoTRX6*	A1.8G025600	chr8a1	121	13.24	4.95	3	Nucleus	c1xflA	Thioredoxin h1
*CoTRX7*	A1.9G067200	chr9a1	189	21.15	9.05	6	Chloroplast	c6g62A	Thioredoxin O2
*CoTRX8*	A1.15G023400	chr15a1	79	9.09	4.6	4	Extracellular space	c3d22A	Thioredoxin H-type
*CoTRX9*	A2.1G086600	chr1a2	149	17.31	9.27	4	Endomembrane system	c6x0bA	Thioredoxin H
*CoTRX10*	A2.4G068900	chr4a2	115	12.84	5.65	3	Nucleus	c1xflA	Thioredoxin h1
*CoTRX11*	A2.6G043700	chr6a2	93	10.28	9.82	3	Chloroplast	c6x0bA	Thioredoxin H
*CoTRX12*	A2.6G201400	chr6a2	85	9.30	6.24	2	Chloroplast	c6x0bA	Thioredoxin H
*CoTRX13*	A2.8G139200	chr8a2	191	20.84	8.77	2	Chloroplast	c8qpdA	Thioredoxin M-type
*CoTRX14*	B1.2G091900	chr2b1	189	20.95	8.57	6	Chloroplast	c6g61A	Thioredoxin O1
*CoTRX15*	B1.2G117300	chr2b1	180	20.29	5.15	4	Chloroplast	c8w9zP	Thioredoxin-like CITRX1
*CoTRX16*	B1.4G234200	chr4b1	140	15.34	6.42	3	Chloroplast	c6x0bA	Thioredoxin H
*CoTRX17*	B1.5G132500	chr5b1	172	19.15	9.14	2	Chloroplast outer membrane	c8qpdA	Thioredoxin M-type
*CoTRX18*	B1.6G199900	chr6b1	185	19.86	9.36	2	Chloroplast outer membrane	c8qpdA	Thioredoxin M-type
*CoTRX19*	B1.7G135100	chr7b1	181	19.70	9.35	3	Chloroplast outer membrane	c7c2bC	Thioredoxin F2
*CoTRX20*	B2.11G006300	chr11b2	179	20.25	8.96	2	Chloroplast	c5vo7A	Thioredoxin
*CoTRX21*	C2.4G306700	chr4c2	139	15.53	4.98	3	Chloroplast thylakoid membrane	c6x0bA	Thioredoxin H
*CoTRX22*	C2.5G224200	chr5c2	98	10.97	5.17	4	Mitochondrion	c7bzkB	Thioredoxin Y1
*CoTRX23*	C2.7G132800	chr7c2	85	9.30	6.24	2	Chloroplast	c6x0bA	Thioredoxin H
*CoTRX24*	C2.7G235200	chr7c2	122	14.04	9.96	2	Chloroplast outer membrane	c8qpdA	Thioredoxin M-type
*CoTRX25*	C2.10G026400	chr10c2	172	19.30	9.07	4	Cytoplasm	c3d22A	Thioredoxin H-type
*CoTRX26*	C2.15G002900	chr15c2	85	92.97	6.24	2	Extracellular space	c3d22A	Thioredoxin H-type
*CoTRX27*	C2.15G092100	chr15c2	191	20.65	9.36	2	Chloroplast	c6x0bA	Thioredoxin H

**Table 2:** The *K_a_*, *K_s_* and *ω* between *CoTRX* paralog pairs of *Camellia oleifera*.

Paralog Pairs		Duplication	*K_a_*	*K_s_*	*ω* (*K_a_/K_s_*)
		Type			
CoTRX25.1	CoTRX3.1	Segmental	0.0961	0.2755	0.3487
CoTRX1.1	CoTRX4.1	Segmental	0.2312	1.4515	0.1593
CoTRX1.1	CoTRX6.1	Segmental	0.2412	0.9251	0.2608
CoTRX1.1	CoTRX10.1	Segmental	0.1554	0.6061	0.2564
CoTRX4.1	CoTRX10.1	Segmental	0.1614	1.0115	0.1596
CoTRX4.1	CoTRX6.1	Segmental	0.1004	0.6293	0.1595
CoTRX6.1	CoTRX10.1	Segmental	0.1648	1.1239	0.1467
CoTRX18.1	CoTRX24.1	Segmental	0.1027	0.5517	0.1861
CoTRX3.2	CoTRX25.2	Segmental	0.1135	0.3081	0.3684
CoTRX18.2	CoTRX24.2	Segmental	0.0987	0.4328	0.2281
CoTRX1.3	CoTRX4.3	Segmental	0.2820	1.4789	0.1907
CoTRX1.3	CoTRX6.3	Segmental	0.2704	0.9981	0.2709
CoTRX3.3	CoTRX25.3	Segmental	0.0961	0.2902	0.3313
CoTRX4.3	CoTRX6.3	Segmental	0.1004	0.5743	0.1748
CoTRX4.3	CoTRX10.3	Segmental	0.3152	1.2577	0.2507
CoTRX18.3	CoTRX24.3	Segmental	0.1644	0.6501	0.2528
CoTRX1.4	CoTRX4.4	Segmental	0.2898	1.3189	0.2197
CoTRX1.4	CoTRX6.4	Segmental	0.2726	0.9843	0.2770
CoTRX1.4	CoTRX10.4	Segmental	0.1980	0.5525	0.3585
CoTRX3.4	CoTRX25.4	Segmental	0.1175	0.3071	0.3827
CoTRX4.4	CoTRX6.4	Segmental	0.1308	0.5757	0.2271
CoTRX1.5	CoTRX4.5	Segmental	0.2309	1.2991	0.1778
CoTRX1.5	CoTRX6.5	Segmental	0.2433	0.9142	0.2661
CoTRX1.5	CoTRX10.5	Segmental	0.1598	0.6415	0.2491
CoTRX3.5	CoTRX25.5	Segmental	0.3167	0.7476	0.4237
CoTRX4.5	CoTRX10.5	Segmental	0.1640	1.0816	0.1516
CoTRX6.5	CoTRX10.5	Segmental	0.1670	1.1601	0.1440
CoTRX18.5	CoTRX24.5	Segmental	0.1105	0.4981	0.2219
CoTRX1.6	CoTRX6.6	Segmental	0.2418	0.9597	0.2519
CoTRX1.6	C2.4G094900	Segmental	0.1554	0.6370	0.2439
CoTRX3.6	CoTRX25.6	Segmental	0.1121	0.2685	0.4174
CoTRX6.6	C2.4G094900	Segmental	0.1652	1.1667	0.1416
CoTRX13.6	CoTRX22.6	Segmental	0.7456	NaN	NaN
C2.6G214900	CoTRX24.6	Segmental	0.1071	0.5110	0.2096

## Data Availability

The original contributions presented in this study are included in the Supplementary Material. Further inquiries can be directed to the corresponding author(s).

## Supplementary Materials

The following supporting information can be downloaded at: https://www.mdpi.com/article/10.3390/genes16070790/s1, Table S1: The orthologous genes of typical TRXs in *Arabidopsis*, rice and tea tree; Table S2: PCR using specific primers; Table S3: The distribution of CoTRXs on oil-Camellia chromosomes across 6 different haplotypes; Table S4: FPKM values of *CoTRXs* in various tissues and embryo development.
